# Changes in Voluntary Activation Assessed by Transcranial Magnetic Stimulation during Prolonged Cycling Exercise

**DOI:** 10.1371/journal.pone.0089157

**Published:** 2014-02-21

**Authors:** Marc Jubeau, Thomas Rupp, Stephane Perrey, John Temesi, Bernard Wuyam, Patrick Levy, Samuel Verges, Guillaume Y. Millet

**Affiliations:** 1 INSERM U1042, Grenoble, France; 2 Université de Lyon, Saint-Etienne, France; 3 MIP, Nantes, France; 4 Laboratoire HP2, Grenoble Alpes University, Grenoble, France; 5 Movement To Health (M2H), Montpellier-I University, Euromov, France; 6 Human Performance Laboratory, Faculty of Kinesiology, University of Calgary, Calgary, Canada; McGill University, Canada

## Abstract

Maximal central motor drive is known to decrease during prolonged exercise although it remains to be determined whether a supraspinal deficit exists, and if so, when it appears. The purpose of this study was to evaluate corticospinal excitability and muscle voluntary activation before, during and after a 4-h cycling exercise. Ten healthy subjects performed three 80-min bouts on an ergocycle at 45% of their maximal aerobic power. Before exercise and immediately after each bout, neuromuscular function was evaluated in the *quadriceps femoris* muscles under isometric conditions. Transcranial magnetic stimulation was used to assess voluntary activation at the cortical level (VA_TMS_), corticospinal excitability via motor-evoked potential (MEP) and intracortical inhibition by cortical silent period (CSP). Electrical stimulation of the femoral nerve was used to measure voluntary activation at the peripheral level (VA_FNES_) and muscle contractile properties. Maximal voluntary force was significantly reduced after the first bout (13±9%, P<0.01) and was further decreased (25±11%, P<0.001) at the end of exercise. CSP remained unchanged throughout the protocol. *Rectus femoris* and *vastus lateralis* but not *vastus medialis* MEP normalized to maximal M-wave amplitude significantly increased during cycling. Finally, significant decreases in both VA_TMS_ and VA_FNES_ (∼8%, P<0.05 and ∼14%, P<0.001 post-exercise, respectively) were observed. In conclusion, reductions in VA_FNES_ after a prolonged cycling exercise are partly explained by a deficit at the cortical level accompanied by increased corticospinal excitability and unchanged intracortical inhibition. When comparing the present results with the literature, this study highlights that changes at the cortical and/or motoneuronal levels depend not only on the type of exercise (single-joint vs. whole-body) but also on exercise intensity and/or duration.

## Introduction

Neuromuscular fatigue - defined as strength loss - may develop during exercise and its etiology depends on several factors such as the intensity and duration of exercise. The effects of exercise of 30 min or longer in duration, mostly human locomotion such as running, cycling and cross-country skiing, on neuromuscular function have been extensively investigated over the last 10 years [1]–[5]. Fatigue can originate at several potential sites that are usually classified as being proximal (central fatigue) or distal (peripheral fatigue) to the neuromuscular junction. Since this distinction was first considered in 1931 by Bainbridge [6], much more has been learned about central and peripheral origins. It is now well-understood that such origins are mutually dependent as the recruitment of motoneurons depends on the descending drive from supraspinal sites and central drive is controlled through a combination of influences including excitatory and inhibitory reflexes [7]. Although non-normalized EMG must be interpreted with caution when used to assess central drive, it has been shown that a decrease in intramuscular pH is associated with declines in maximal strength (i.e. fatigue) and integrated electromyography (EMG), illustrating a feedback mechanism inhibiting maximal central motor drive [8].

It has been systematically shown that central fatigue largely contributes to strength loss after prolonged running exercise [9]. Central fatigue is generally lower after prolonged cycling exercise, having been detected in some [1], [10], [11] but not all [12], [13] studies. By measuring an activation deficit with the twitch interpolation technique at the peripheral nerve or muscle levels or using normalized EMG during isometric maximal voluntary contractions (MVC), it is not possible to determine if the central fatigue originates from a supraspinal site and/or at the spinal level [14]. It has been suggested that neurally-mediated afferent feedback from the muscle (i.e. presynaptic inhibition and fusimotor system disfacilitation) contributes, among other factors, to inhibition of motoneuronal excitability [5]. However, group III and IV afferent fibers do not only act at the motoneuronal level. For instance, Gandevia et al. [15] showed that the increment of force (superimposed twitch, SIT) evoked by transcranial magnetic stimulation (TMS) increases during a sustained MVC, suggesting the development of supraspinal fatigue. Interestingly, when the muscle was held ischemic after the sustained MVC, VA assessed by TMS (VA_TMS_) remained impaired while fatigue-induced changes in EMG responses to TMS (i.e. motor-evoked potential, MEP and cortical silent period, CSP; see below) recovered rapidly. This suggests that a supraspinal failure, i.e. a decrease in VA_TMS_, differs from an impaired corticospinal excitability.

This experiment [15] was among the first to use TMS and measure both mechanical and EMG responses in the context of fatigue. The same research group proposed a method to measure VA_TMS_. This method is based on the classical interpolated twitch technique [16], except that the resting twitch is calculated by linear extrapolation of the regression between the superimposed force evoked by TMS and voluntary force at 50%, 75% and 100% MVC [17], [18]. Originally applied in the elbow flexors, the validity and reliability of this technique was ascertained for other muscle groups such as quadriceps [19], [20]. Based on this method, two studies recently documented significant decreases in VA_TMS_ (∼10%) [10], [21] after cycling exercise conducted at ∼80% of maximal aerobic power (W_max_), i.e. lasting 8 to 40 min. Recently, we observed a tendency toward significant decrease in VA_TMS_ [22] after a lower intensity progressive protocol lasting ∼60 min. Another study did not observe any change 10 min post-exercise after ∼90 min cycling exercise [23]. It is unknown whether a supraspinal deficit occurs throughout longer cycling exercise and if so, the kinetics of supraspinal fatigue appearance and whether or not this is associated with a decrease in corticospinal excitability remain to be determined. Therefore, the aim of this study was to test the hypotheses that (i) prolonged cycling exercise decreases VA_TMS_ and (ii) this central fatigue occurs despite unchanged or elevated MEP responses. For this purpose, corticospinal excitability, intracortical inhibition and voluntary activation assessed by both peripheral electrical stimulation and TMS were evaluated before and after a 4-h cycling exercise and at regular intervals during the exercise session.

## Materials and Methods

### Subjects

Ten healthy male subjects participated in this study. All subjects were non-smokers and had no history of cardiorespiratory or neuromuscular disease. They reported training between 3 and 12 h per week, mostly in aerobic-type activities. Their mean (± SD) age, stature and mass were 37.3±7.3 yr, 180.2±5.2 cm, 73.1±7.0 kg, respectively. Subjects refrained from physical exercise on the 2 d prior to the tests, abstained from drinking caffeinated beverages on test days, slept for at least 7 h the night before the tests and had their last meal at least 2 h prior to the tests.

### Ethics Statement

The experiments were conducted according to the Declaration of Helsinki. Subjects were fully informed of the procedure and risks involved and gave their written consent. They were also familiarized with all experimental procedures used in the present study, including magnetic and electrical stimulation. Approval for the experiment was obtained from the local Committee on Human Research (Comité de protection des personnes Sud-EST V, 2010-A00121-38).

### Experimental Design

The effects of prolonged cycling on neuromuscular fatigue, particularly central fatigue, were assessed in the right *quadriceps femoris* muscle under isometric conditions. TMS was used to assess motor corticospinal excitability and intracortical inhibition via MEP and CSP measurements, respectively. Femoral nerve electrical stimulation (FNES) was also performed to measure neuromuscular transmission and contractile properties. VA was assessed with both FNES and TMS (see below). EMG of *vastus lateralis* (VL), *rectus femoris* (RF) and *vastus medialis* (VM) was measured continuously during cycling. Subjects rested for at least 20 min before the first measurements (PRE), permitting skin preparation and surface EMG electrode placement.

**Figure 1:**
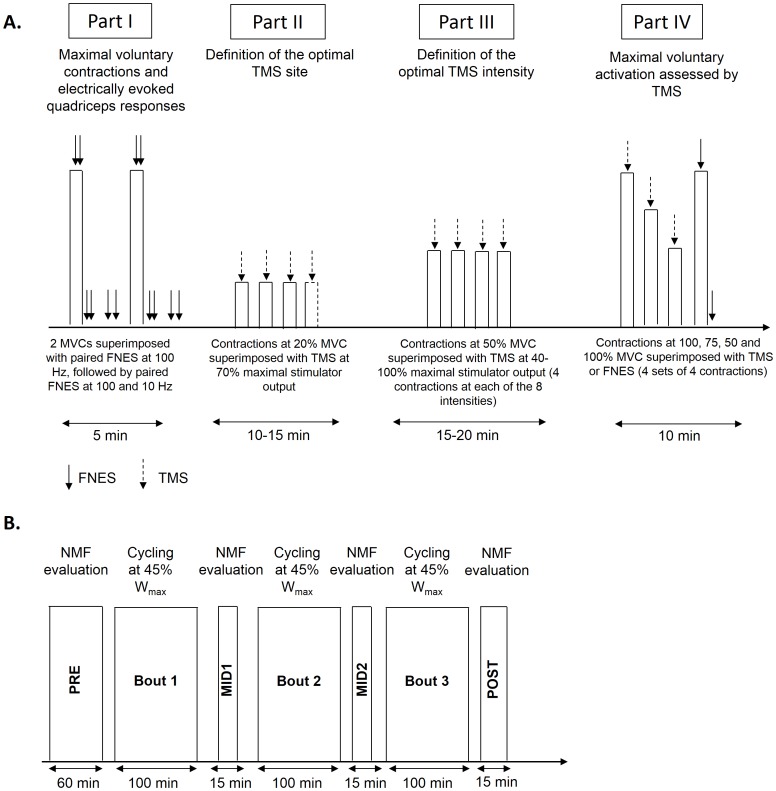
Overview of the neuromuscular function (NMF) evaluation (panel A) and the experimental protocol (panel B). NMF evaluation before exercise (PRE) consisted of four parts in the following order: Part I: MVC and electrically evoked quadriceps responses; Part II: determination of the optimal TMS site; Part III: determination of the optimal TMS intensity; Part IV: maximal voluntary activation with TMS. NMF consisted in part I and IV only after the first (MID1), the second (MID2) and the third (POST) bout of cycling.

After EMG electrode placement and determination of FNES intensity, the testing protocol summarized in Figure 1A was performed. At PRE, this consisted of: I) measurement of maximal voluntary force and electrically-evoked quadriceps responses: subjects performed ∼10 submaximal warm-up knee extensions followed by 2 (or 3 at PRE only if the first two differed by more than 5%) MVCs with supramaximal FNES paired-pulses delivered during and immediately following the MVC to assess VA_FNES_ and knee extensor contractile properties, II) determination of the optimal TMS site, III) determination of the optimal TMS intensity (recruitment curve) and IV) sets of voluntary activation to assess VA_TMS_, MEP and CSP. The neuromuscular function testing after the first (MID1), second (MID2) and third (POST) 80-min bout of cycling consisted of only parts I (without warm-up) and IV (see Fig. 1B). The neuromuscular testing lasted for ∼60 min at PRE and 15 min at MID1, MID2 and POST and started ∼3 min after the cessation of each cycling bout.

#### Cycling exercise

Each subject completed a progressive cycling exercise test to exhaustion at least 1 week before the protocol. The test was performed on a computer-controlled electrically-braked cycle ergometer (Corival, Lode, Groningen, The Netherlands) and started at 90 W, increasing by 15-W increments every minute until volitional exhaustion. Maximal power output (i.e. the highest power output sustained for at least one minute) and maximal oxygen uptake (Medisoft, Dinant, Belgium) were determined (339±51 W and 59±8 ml•min^−1^•kg^−1^, respectively).

To evaluate the effects of prolonged cycling on neuromuscular fatigue during a second visit, each subject performed three 80-min cycling bouts at 45% W_max_ at a self-selected pedaling rate separated by 15 min of neuromuscular testing (Fig. 1B). During the 4-h cycling exercise, subjects consumed a 70 g·L^−1^ glucose polymer solution (2220±460 ml) and energy cakes (n = 1.5±1.3) (Go2, Chartres de Bretagne, France) *ad libitum*.

### Dependent Variables

#### Force measurements

Subjects sat on a custom-built quadriceps chair with the right hip angle set at 90° and knee joint angle set at 100° of flexion (full knee extension is at 180°). A noncompliant strap connected to a strain gauge (DEC60, Captels, St Mathieu de Treviers, France) was attached around the subject’s shank, 3–5 cm above the lateral malleoli. Force data were acquired using a PowerLab data acquisition system (16/30 - ML880/P, ADInstruments, Bella Vista, Australia) at a sampling rate of 2 kHz. The subjects were firmly secured to the chair with noncompliant straps to minimize body movement.

#### Electrical nerve stimulation

Electrical stimulation was delivered percutaneously to the femoral nerve via a self-adhesive electrode manually pressed by an experimenter into the femoral triangle to minimize the stimulus intensity and discomfort (10-mm diameter, Ag-AgCl, Type 0601000402, Contrôle Graphique Medical, Brie-Comte-Robert, France). The anode, a 10×5 cm self-adhesive stimulation electrode (Medicompex SA, Ecublens, Switzerland), was located in the gluteal fold. A constant current stimulator (Digitimer DS7A, Hertfordshire, United Kingdom) was used to deliver a square-wave stimulus of 1-ms duration with maximal voltage of 400 V. The optimal stimulus intensity was determined from maximal twitch force measurement (see below). The stimulating intensity (135±33 mA) was supramaximal, i.e. 150% of optimal intensity. Supramaximal FNES was delivered: i) during MVCs (paired high-frequency stimuli at 100 Hz, see part I of neuromuscular testing in Fig. 1A), ii) 2 s after the MVCs in relaxed muscle as paired high- and low-frequency (10 Hz) stimuli separated by a 5-s interval (Fig. 1A, part I) and iii) during and 2 s after the last MVC of the 4-contraction sets as single-pulse FNES to obtain M wave (Fig. 1A, part IV).

#### Transcranial magnetic stimulation

A magnetic stimulator (Magstim 200, The Magstim Company, Dyfed, UK) was used to stimulate the motor cortex. Single TMS pulses of 1-ms duration were delivered via a concave double-cone coil (diameter: 110 mm; maximum output: 1.4 T) positioned over the vertex of the scalp and held tangentially to the skull. The coil was positioned to preferentially activate the left motor cortex (contralateral to the right leg) and elicit the largest MEP in the RF and the VL with only a small MEP in the *biceps femoris* during isometric knee extension at 20% MVC with a stimulus intensity of 70% of maximal stimulator power output [20]. The optimal stimulus site was marked on a white hypoallergenic tape which was taped directly to the scalp to ensure reproducibility of the stimulus conditions for each subject throughout the entire protocol. After 3 min of rest, TMS during brief (∼5 s) isometric knee extensions at 50% MVC, i.e. the force level inducing the largest MEP [20], were performed at 40, 50, 60, 70, 80, 90 and 100% of maximal stimulator power output in random order. Four consecutive contractions were performed at each stimulus intensity with 10 s between contractions at the same stimulation intensity and 30 s between series of 4 contractions. The stimulus intensity (58±9% of stimulator maximal power output) that elicited the largest right RF MEPs with small MEP of the right *biceps femoris* (amplitude <10% of maximal RF M-wave) was considered optimal and employed throughout the protocol, as previously suggested [24]. After another 3 min of rest, VA assessment consisted of 4 sets of 4 brief (∼2 s) contractions at 100%, 75%, 50% (calculated from the first MVC of each set) and 100% MVC with 10 s of rest between contractions and 30 s between series [20]. TMS was delivered during the first three contractions and FNES (single stimulus) was delivered during and 2 s after the last contraction (Fig. 1A, part IV). Strong verbal encouragement was given during MVCs and real-time visual feedback of target force levels were provided via custom software (Labview 8, National Instrument, Austin, TX) on a computer screen throughout the experiment.

#### Electromyographic recordings

The EMG signals of the right VL, VM and RF and *biceps femoris* were recorded using bipolar silver chloride surface electrodes of 10-mm diameter (Type 0601000402, Contrôle Graphique Medical) during the voluntary contractions and electrical/magnetic stimuli. The recording electrodes were secured lengthwise to the skin over the muscle belly following SENIAM recommendations [25], with an interelectrode distance of 25 mm. The position of the electrodes was marked on the skin in case replacement was necessary. The reference electrode was attached to the patella. Low impedance (Z <5 kΩ) at the skin-electrode surface was obtained by abrading the skin with fine sand paper and cleaning with alcohol. EMG data were recorded with a PowerLab system (16/30 - ML880/P, ADInstruments) with a sampling frequency of 2 kHz. The EMG signal was amplified with octal bio-amplifier (Octal Bioamp, ML138, ADInstruments) with a bandwidth frequency ranging from 5 to 500 Hz (input impedance = 200 MΩ, common mode rejection ratio = 85 dB, gain = 1,000), transmitted to the PC and analyzed with LabChart 7 software (ADInstruments).

### Data Analysis

#### Mechanical responses

Peak forces measured during MVC were calculated as the maximal values obtained before the stimuli during the two contractions (Fig. 1A, part I) performed at PRE, while the first MVC was used solely at MID1, MID2 and POST to reduce the influence of fatigue recovery. The amplitude of the potentiated high-frequency doublet (P_Db100_, Fig. 1A, part I), ratio of peak forces from paired low- and high-frequency stimuli (Db10∶100) to assess low-frequency fatigue [26] and amplitude of the potentiated resting twitch (Pt) (Fig. 1A, part IV) were also determined. For P_Db100_ and Db10∶100, the value of the first set was considered (Fig. 1A, part I). For Pt, the mean value was computed from the four sets (Fig. 1A, part IV).

#### M-wave

M-wave peak-to-peak amplitude and duration were calculated from EMG responses to single FNES in the relaxed and contracting muscles, with the mean value computed from the four sets (Fig. 1A, part IV).

#### VA_FNES_

Electrical stimuli were delivered during MVCs to evaluate VA_FNES_. The technique was adapted from the twitch interpolation technique and consists of delivering a high-frequency paired-pulse at supramaximal intensity during the isometric force plateau. In the present study, the stimulation was delivered at 98.4±2.0%, 97.2±2.2%, 98.0±1.4% and 96.8±2.5% of MVC force at PRE, MID1, MID2 and POST, respectively, indicating the validity of our methodology. The control high-frequency paired-pulse was delivered in the relaxed muscle, 2 s after the end of the 5-s contraction. This elicits a potentiated mechanical response to ensure that the muscle is tested in the same state. The ratio of the amplitude of the superimposed doublet to the amplitude of the control doublet was then calculated to obtain VA_FNES_ as follows:

VA_FNES_ = [1 - (Superimposed Db100/P_Db100_)]×100.

#### TMS parameters

Peak-to-peak MEP amplitudes of quadriceps muscles during TMS delivered during submaximal and maximal contractions were normalized to peak-to-peak maximal M-wave amplitudes elicited by single FNES delivered during the MVC (M_sup_) [39]. The duration of the CSP was determined manually as the interval from the stimulus to the return of continuous EMG activity [21].

VA_TMS_ was quantified by measurement of the superimposed force responses to TMS. Because motor cortical and spinal cord excitability increase during voluntary contractions, it is necessary to estimate rather than directly measure the amplitude of the resting twitch evoked by motor-cortical TMS [17]. The mean SIT amplitude evoked during contractions at 100, 75, and 50% MVC was calculated for the four sets, and the y-intercept of the linear regression between the mean SITs and voluntary force was used to quantify the estimated resting twitch (ERT) [17], [19], [20]. VA_TMS_ (%) was then calculated using the following equation:

VA_TMS_ = [1 - (SIT/ERT)] ×100.

The reliability of this method for the determination of VA_TMS_ has been previously described for the knee extensors [19], [20].

#### EMG during cycling

During the three bouts of cycling, EMG parameters were analyzed over 3-min periods at the beginning of each bout and every 20 min (i.e. +20, +40, +60 and +80 min). The root-mean-square (RMS) of the 3-min periods (CYCL_RMS/M_) was calculated for each muscle (VL, RF, VM). Then EMG RMS was normalized to the maximal M-wave amplitude recorded before the bout, i.e. PRE for bout 1, MID1 for bout 2 and MID2 for bout 3.

#### Blood lactate, blood glucose and core temperature

A small capillary blood sample (5 µL) was obtained from the finger to measure blood glucose (ACCU-CHEK Performa, Roche Diagnostics, Mannheim, Germany) and lactate (Lactate Plus, Nova Biomedical Corporation, Waltham, MA) concentrations. Core temperature (infrared ear thermometer, Braun ThermoScan, Type 6021, Kaz Europe SA, Lausanne, Switzerland) was also measured at the beginning (PRE) and in the last minute of each 80-min cycling bout.

#### Perceived exertion

The subjects were asked to report their rating of perceived exertion (RPE), i.e., how hard they perceived the exercise, at the beginning of each bout and every 20 min during the three cycling bouts using a 100-mm visual analog scale, with “no effort” on one end (0 mm) and “exhaustion” on the other (100 mm) [27], [28].

### Statistical Analyses

All statistics presented are mean values ± SD. The assumption of normality for all target variables was assessed using the Kolmogorov-Smirnov test. The assumption of sphericity was assessed with Mauchly’s test, and in the case of significant violations, the Greenhouse-Geisser correction was applied. For MVC, P_Db100_, Db10∶100, Pt, ERT, VA_TMS_, VA_FNES_ and M-wave amplitude and duration, a one-way (PRE-MID1-MID2-POST) ANOVA with repeated measures was performed to assess the effect of exercise duration. MEP normalized to M_sup_ and CSP were analyzed for each muscle (VL, RF, VM) by two-way ANOVA (%MVC×time) with repeated measures for the 3 intensities (50, 75 and 100% MVC) and 4 time points (PRE-MID1-MID2-POST). For RPE and EMG parameters measured during cycling, a two-way ANOVA (bout×time) with repeated measures was performed for the 3 bouts and 5 time points of each bout (0, +20, +40, +60, +80 min) to analyze the kinetics of changes due to prolonged exercise. Changes in blood glucose, blood lactate and core temperature over time were also assessed with a one-way (PRE-bout1-bout2-bout3) ANOVA with repeated measures. Where the ANOVA revealed significant main effects or interactions, the data were further explored using pair-wise comparisons with Bonferroni correction. Relationships between selected parameters were determined by Pearson product correlation. For all statistical analyses, an alpha level of 0.05 was used as the cut-off for significance.

## Results

**Figure 2:**
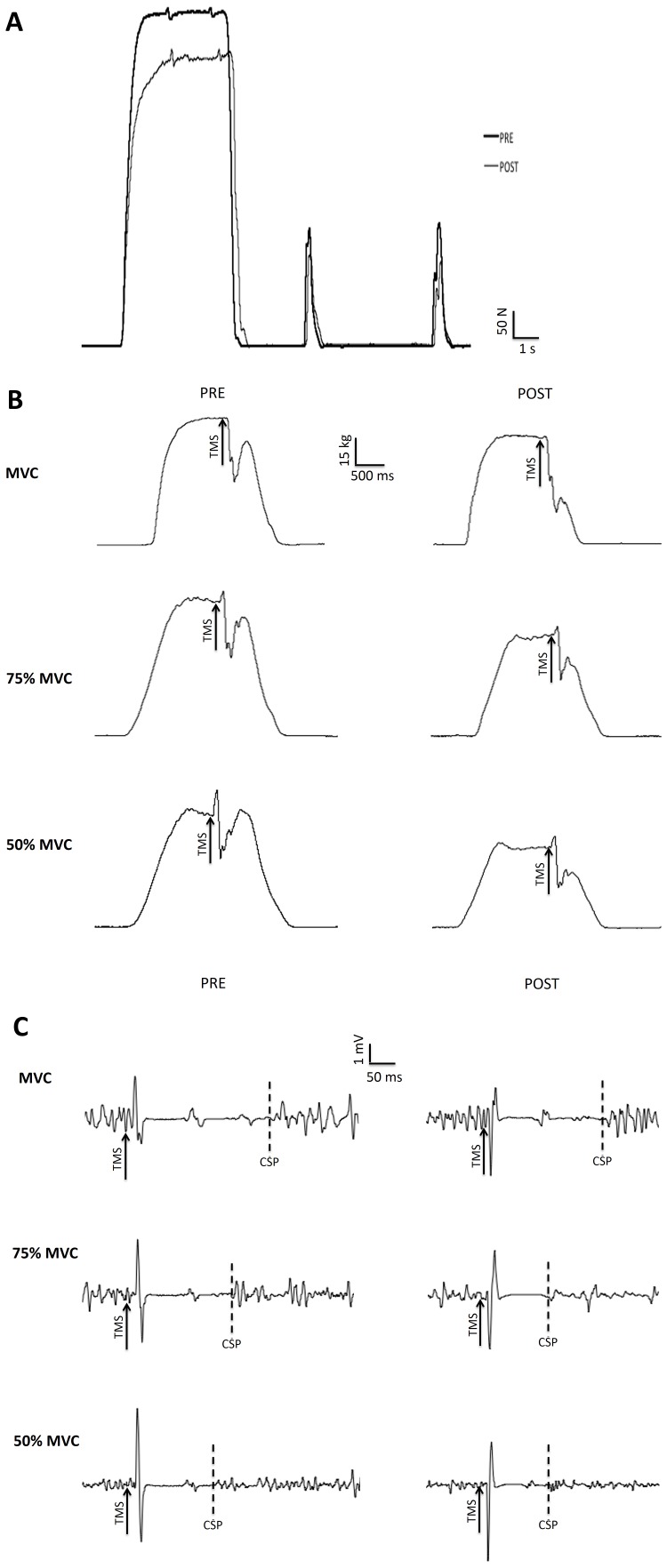
Typical traces recorded before (Pre) and after (Post) the 4-h cycling bout of exercise for MVC force and potentiated doublets (panel A), maximal voluntary activation assessed with TMS during contractions at 100%, 75% and 50% MVC (panel B), and motor evoked potential for the *rectus femoris* muscle recorded during contractions at 100%, 75% and 50% of MVC (panel C). Arrows indicates the time of transcranial magnetic stimulation (TMS). Dotted lines indicate the end of the cortical silent period (CSP).

Figure 2 shows typical traces of MVC and potentiated doublets (2A), maximal voluntary activation assessed with TMS (2B), and MEP (2C) for a single subject before and after the 4-h cycling bout of exercise.

### MVC and Peripheral Fatigue

**Figure 3:**
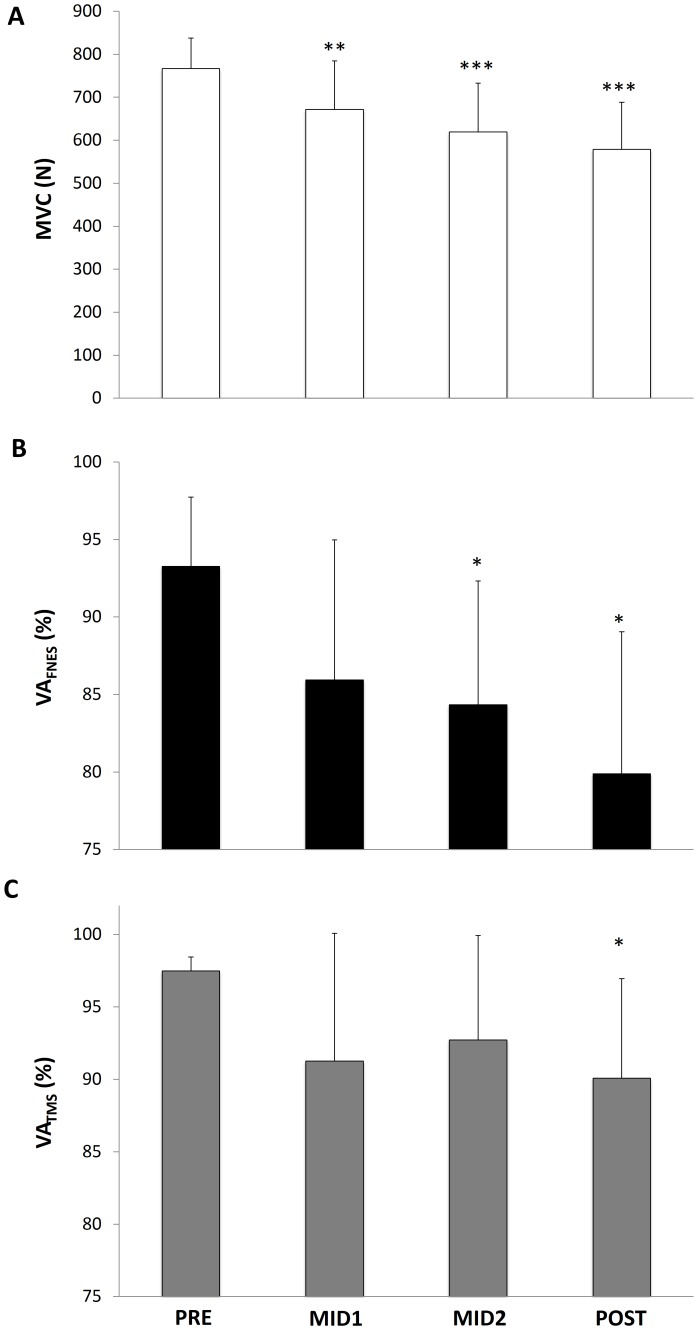
Maximal voluntary contraction (MVC) force (panel A), maximal voluntary activation assessed by interpolated twitch technique (VA_FNES_, panel B) and maximal voluntary activation assessed by transcranial magnetic stimulation (VA_TMS_, panel C) of the knee extensor muscles, before (PRE), after 80 min (MID1) and 160 min (MID2) of cycling and after the 4-h cycling exercise (POST). Significantly different from PRE: *P<0.05, **P<0.01, ***P<0.001.

MVC decreased linearly over time (P<0.001; Fig. 3A). Indeed, MVC was significantly reduced at MID1 (by 13±9%, P<0.01), and further reduced at MID2 (by 20±11%, P<0.001) and POST (by 25±11%, P<0.001). P_Db100_ was also significantly depressed after the first bout (by 20±8%, P<0.001, Table 1) and remained stable thereafter. Db10∶100 evolved in a similar manner to P_Db100_, i.e. showing a significant reduction after the first bout (by 23±14%, P<0.01) and remaining constant thereafter (Table 1). Mechanical and EMG responses to single-pulse FNES and ERT are presented in Table 1.

**Table 1:** Amplitude of the potentiated high-frequency paired-pulse force (P_Db100_), potentiated peak twitch force (Pt), estimated resting twitch (ERT) and ratio of paired-pulse peak forces at 10 Hz to 100 Hz (Db10∶100) in the knee extensor muscles, and EMG (M-wave) responses to single-pulse stimuli for *rectus femoris* (RF), *vastus lateralis* (VL) and *vastus medialis* (VM) muscles before (PRE), after 80 min (MID1) and 160 min (MID2) of cycling and after the 4-h cycling exercise (POST).

		PRE	MID1	MID2	POST
P_Db100_ (N)		281±20	224±27***	223±23***	215±19***
Pt (N)		171±26	137±31**	131±30**	123±29**
ERT (N)		119±35	90±35*	82±31*	75±25**
Db10∶100		1.04±0.09	0.79±0.14**	0.81±0.15*	0.75±0.16**
	RF	9.0±2.2	7.2±2.6**	7.2±2.3***	7.5±2.3*
M-wave amplitude (mV)	VL	16.4±3.0	13.7±4.5	13.3±4.3*	12.3±5.2
	VM	12.8±3.5	10.3±3.6	10.1±3.5*	11.2±2.9
	RF	9.9±1.6	10.7±2.7	10.5±2.6	9.7±2.0
M-wave duration (ms)	VL	8.3±1.1	8.9±2.0	9.4±1.4***	10.2±1.5**
	VM	8.6±1.5	10.8±3.4*	12.7±2.9*	13.0±3.1**

Data are mean values ± SD. Significantly different from PRE: *P<0.05, **P<0.01, ***P<0.001.

### Voluntary Activation and Corticospinal Excitability

**Figure 4:**
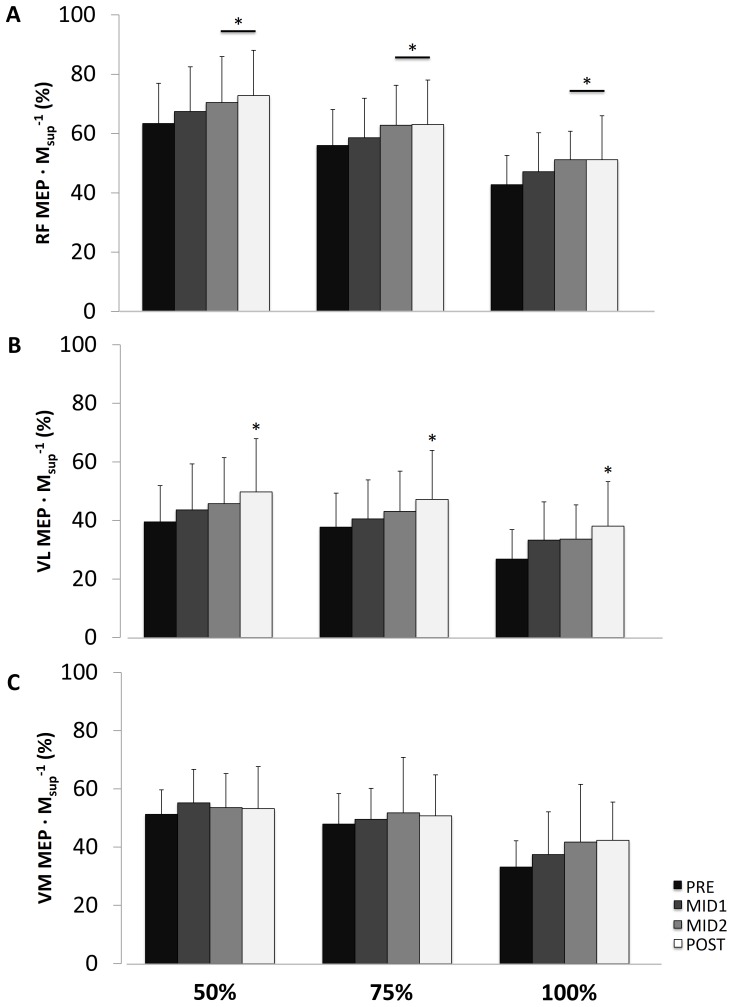
Motor-evoked potential normalized to maximal M-wave amplitude (MEP·M_sup_^−1^) at 50, 75 and 100% of maximal voluntary contraction level for *rectus femoris* (RF, panel A), *vastus lateralis* (VL, panel B) and *vastus medialis* (VM, panel C) before (PRE), after 80 min (MID1) and 160 min (MID2) of cycling and after the 4-h cycling exercise (POST). Significantly different from PRE: *P<0.05.

VA_FNES_ and VA_TMS_ decreased over time (P<0.01 and P<0.05, respectively) (Fig. 3B and 3C, respectively). As shown in Figures 4A and 4B, TMS delivered during submaximal and maximal contractions elicited RF MEP·M_sup_^−1^ that were significantly greater at MID2 and POST (P<0.05) compared to PRE. VL MEP·M_sup_^−1^ was also greater at POST (P<0.05) compared to PRE whereas no significant time effect was noted for VM MEP·M_sup_^−1^ (Fig. 4C). Mean CSPs were similar over time (Table 2).

**Table 2:** Corticospinal silent period (ms) of the *rectus femoris* (RF), *vastus lateralis* (VL) and *vastus medialis* (VM) during contractions at 50, 75 and 100% of maximal voluntary contraction (MVC) of the *quadriceps femoris* muscles before (PRE), after 80 min (MID1) and 160 min (MID2) of cycling and after (POST) the 4-h cycling exercise.

	RF				VL				VM			
	PRE	MID1	MID2	POST	PRE	MID1	MID2	POST	PRE	MID1	MID2	POST
50% MVC	175±62	171±62	174±64	173±64	175±64	171±63	177±66	173±65	171±69	170±66	168±72	170±65
75% MVC	167±72	172±71	171±70	167±66	165±73	166±72	169±72	163±68	164±73	163±73	164±77	162±69
100% MVC	164±83	165±85	167±79	166±79	161±86	160±86	164±81	162±77	159±82	158±81	159±85	161±79

Data are mean ± SD.

### EMG Parameters and RPE During Cycling

Pedaling rate remained constant throughout the entire protocol (77±12 rpm). As shown in Table 3, RPE increased linearly over time (P<0.001). RPE was significantly greater during bouts 2 and 3 compared to bout 1 (P<0.01 and P<0.001, respectively). No significant modifications of RF, VL and VM Cycl_RMS/M_ were observed during cycling over time (Table 3).

**Table 3:** Total EMG root-mean-square (RMS) normalized by maximal M-wave amplitude (Cycl_RMS/M_) measured at the beginning of each bout and every 20 min during the three 80-min bouts of cycling for the *rectus femoris* (RF), *vastus lateralis* (VL) and *vastus medialis* (VM) muscles and rating of perceived exertion (RPE), blood glucose, blood lactate and core temperature measured before (PRE) the 4-h cycling exercise and at the end of every 80 min cycling bout.

	PRE	Bout 1					Bout 2					Bout 3				
Time (min)		0	20	40	60	80	0	20	40	60	80	0	20	40	60	80
Cycl_RMS/M_ *RF*	/	5.4	5.6	5.7	5.7	5.8	7.2	7.1	7.6	7.4	7.3	6.8	6.7	6.4	6.8	6.4
		±1.8	±1.7	±2.1	±2.2	±2.0	±2.7	±2.4	±3.2	±2.7	±2.8	±2.6	±2.2	±2.4	±2.7	±2.3
Cycl_RMS/M_ *VL*	/	9.3	9.4	9.1	9.2	9.5	12.1	12.6	13.3	13.5	13.6	11.6	12.0	11.4	12.1	11.6
		±2.4	±2.2	±2.1	±2.3	±2.2	±9.5	±9.9	±10.5	±11.2	±10.8	±5.6	±6.3	±5.2	±6.4	±6.0
Cycl_RMS/M_ *VM*	/	14.2	13.9	13.4	13.5	13.7	17.6	18.0	19.4	19.2	19.1	17.1	17.7	17.6	18.4	17.4
		±5.5	±4.6	±4.8	±4.7	±4.5	±11.4	±11.5	±12.7	±12.8	±12.2	±8.5	±8.6	±9.3	±11.1	±9.8
RPE	/	20	20	23	30	37	32**	37**	44**	49**	50**	42***	56***	63***	68***	68***
		±13	±11	±11	±15	±16	±14	±16	±19	±19	±21	±18	±21	±20	±23	±23
Blood glucose	6.6	/	/	/	/	5.3	/	/	/	/	5.0^$$^	/	/	/	/	5.5
(mmol·L^−1^)	±1.2					±0.5					±0.9					±1.1
Blood lactate	1.7	/	/	/	/	1.3	/	/	/	/	1.3	/	/	/	/	1.2
(mmol·L^−1^)	±0.5					±0.4					±0.6					±0.6
Temperature	36.6	/	/	/	/	36.9	/	/	/	/	37.1	/	/	/	/	37.1
(°C)	±0.2					±0.6					±0.6					±0.6

Data are mean values ± SD. Significantly different from PRE: ^$$^ P<0.01. Significant bout effect: Bout1<Bout 2 and 3, **P<0.01 and ***P<0.001.

### Blood Glucose, Blood Lactate and Core Temperature

Blood glucose, blood lactate and core temperature are presented in Table 3. A significant decrease in blood glucose was observed at the end of the second bout (P<0.01) although blood glucose returned to initial values by the end of the third bout. No significant changes were observed for blood lactate and core temperature at any time point during the protocol.

## Discussion

Central fatigue, evaluated by the classical twitch interpolation technique, has been reported to contribute to strength loss after prolonged exercise [5]. The primary goal of the present study was to determine whether at least part of this central fatigue was supraspinal. The main results are that (i) a 4-h cycling exercise did not change cortical silent period duration but increased RF and VL motor-evoked potential amplitude, suggesting elevated corticospinal excitability associated with (ii) a significant decrease in supraspinal fatigue after 240 min of exercise.

### Central and Peripheral Fatigue Time Course During Prolonged Cycling

To the best of our knowledge, only one experiment has examined the time-course of neuromuscular fatigue induced by an acute prolonged cycling exercise [11]. When considering traditional VA, as measured with peripheral stimulation, a reduction in VA_FNES_ comparable to the present study was observed after 5 h of cycling [11] (−8% *vs.* −12% in our experiment). The VA_FNES_ decrease was significant after 160 min in the present study, whereas it only became significant after 4 h of exercise in the study by Lepers et al [11]. During a shorter (<30 min) cycling exercise performed at higher intensity (80% W_max_), Decorte et al. [1] found that VA_FNES_ was significantly decreased only toward the end of exercise. Comparable time courses of central fatigue appearance have been observed during prolonged running [29]–[31]. On the contrary, the main indexes of peripheral fatigue, i.e. knee extensor Pt, P_Db100_ and Db10∶100 and RF, VL and VM M-wave amplitudes, were all significantly reduced at MID1 in the present study. Decreased muscle function at the first time point of measurement has been consistently observed during prolonged cycling exercise [1], [11]. Similar findings were also observed in a more intense exercise involving knee extension [32]. It has been argued that peripheral changes are implicated in the development of central fatigue via type III and IV muscle afferents [7]. These authors have proposed that under normal conditions, such as in the present thermoneutral (environmental temperature: 21.2±0.9°C, relative humidity: 56±16%) and normoxic environment, this neural drive restriction could prevent excessive disturbance of muscle homeostasis and subsequent harm to the body [7]. Our results are not completely in line with this theory. Indeed, the principal changes occurred between PRE and MID1 for muscle function (i.e. changes in P_Db100_, Pt, Db10∶100, and RF, VL and VM M-wave amplitudes) whereas VA alterations statistically developed from MID2, in accordance with the results of Decorte et al. [1]. Alternatively, it is possible that the effects of peripheral fatigue on central drive are delayed. It must be also noted that no correlation was found between changes in VA_TMS_ (or VA_FNES_) and changes in indexes of peripheral fatigue (all R <0.2 and P>0.05), suggesting that central changes might be only partially related to peripheral modifications.

This underlines the necessity to assess neuromuscular function during exercise to further investigate the time course of fatigue etiology and to better understand the potential links between peripheral and central mechanisms and among different central mechanisms (e.g. corticospinal excitability and inhibition, cortical oxygenation (see below), voluntary activation). RPE and central perturbations continued to increase to the end of exercise while peripheral (both EMG and mechanical responses) parameters reached their nadir at MID1 or MID2. A complex combination of feedback and feed-forward mechanisms probably explains this observation [9]. For instance, the increase in RPE observed in the present study may reflect an increase in central motor command, as recently proposed by De Morree et al. [33]. The perception of effort is centrally generated by forwarding corollary discharges or afferent copies [34] from the motor to sensory areas of the cerebral cortex by a corticofugal system. The large increase in the prefrontal cortex oxygenation during the whole exercise [35] could be indicative of a high perception of effort due to cognitive processing required to perform the task and emotional factors, such as the levels of motivation and attention.

### Supraspinal Fatigue During Prolonged Cycling

#### Cortical voluntary activation and motor-evoked potentials

The present study is the first one to assess whether supraspinal fatigue occurs during prolonged cycling exercise. Our results clearly show that this is the case since VA_TMS_ was significantly lower after 4 h of cycling. Ross et al. [36] reported reduced ankle dorsiflexion VA_TMS_, from 75% at baseline to 62% after a marathon, together with depressed MEPs in the *tibialis anterior.* The time-delay between the end of marathon and the testing protocol was relatively long (up to twenty minutes) and, more importantly, the transcranial magnetic stimuli to assess MEP were delivered in relaxed muscle. As recently highlighted [37], TMS evoked at rest complicates interpretation compared to assessing MEP changes in contracted muscles. In particular, MEP variability is lower during contractions than in relaxed muscle and motor cortical excitability is greatly enhanced when muscle is in a contracted state. Thus, it is more appropriate to analyze fatigue-induced MEP changes with TMS delivered during the muscle contraction. More recently, Temesi et al. [38] have also measured VA_TMS_ before and after an ultramarathon running and found a 16% decline.

Three previous studies have used TMS immediately after exercise to examine corticospinal changes due to cycling exercise [10], [21], [22] at much higher exercise intensity (80% W_max_) and of shorter duration than the present one. It is nevertheless worth reporting that these studies measured a significant decrease in VA_TMS_ (∼9–10%) without changes in MEP or CSP responses measured either in VL [10] or RF [21] muscles. Another study did not observe any change in VA_TMS_ and VM/RF MEPs after ∼90 min cycling exercise but the measurements were performed 10 min post-exercise [23]. In our more recent study [22] conducted at a lower exercise intensity and for longer duration although not as prolonged as the present one, a tendency toward decreased VA_TMS_ was observed with an increase in VL MEP. In the present study, normalized RF and VL MEP amplitude increased significantly while VA_TMS_ decreased with fatigue appearance. Similar results were observed for normalized MEP areas (data not shown). The results emphasize that increased corticospinal excitability that generally occurs with fatigue, at least in isometric tasks, can be dissociated from the impairment of voluntary activation [15].

It has been proposed that cardiorespiratory demands (greater during locomotor exercise compared with isometric single-joint exercise) or other factors such as core temperature or blood glucose or catecholamine concentrations could explain the different effects of fatigue on corticospinal excitability between whole-body and single-joint exercises [39]. MEP responses were increased with fatigue in the present study and in Temesi et al. [22] but not after higher-intensity cycling exercise [10], [21]. This shows that intensity/duration of whole-body exercise may also modulate MEP responses, reinforcing the specificity of long-duration exercise previously reported [5]. Liver glycogen depletion and its consequences on blood glucose concentration could in theory explain the difference in corticospinal excitability between short intense and prolonged low-intensity locomotor exercise. However, this is unlikely since in the present study, mean blood glucose concentration remained at or above 5 mmol·L^−1^. Similarly, core temperature remained constant throughout the protocol and could not explain the large changes in corticospinal excitability.

According to Sidhu et al. [39], the mechanisms related to oxygen availability, either originating centrally or from the exercising muscles, may have also contributed to the lack of increase in corticospinal excitability reported in high-intensity whole-body exercise [10], [21], [39]. Exercise intensity was lower (45% W_max_, blood lactate below 2 mmol·L^−1^) in our study compared with exercises conducted at 75–80% W_max_ [10], [21], so that muscle deoxygenation, when considering the exercise-intensity dependence of muscle deoxygenation during exercise [40], was likely attenuated. This probably did not play a major role, if any, in our findings. Of note is that decreased VA_TMS_ has been reported by others to parallel reductions in cerebral O_2_ delivery [10] in the very specific condition of hypoxia. Muscle and cerebral (i.e. prefrontal and motor cortices) NIRS profiles during this 4-h cycling exercise were previously described by our group [41] and demonstrated that even though prefrontal cortex oxygenation is well-preserved during the 4-h cycling exercise, signs of a potential mismatch between oxygen availability and consumption may occur in the motor-related cortical areas, especially during the first bout [41]. In line with the literature [42], these data suggest that central mechanisms of oxygen availability may be linked to perturbations of the central drive from the motor cortex (i.e. decreased VA_TMS_). Further simultaneous TMS and brain metabolism investigations are needed to better understand the precise link between central drive perturbations and mechanisms of cerebral oxygen availability. Sidhu et al. [39] alternatively proposed that the lack of increase in corticospinal excitability found in their whole-body experiments may be related to intrinsic regulatory brain mechanisms associated with an increased internal sense of effort. However, large increases in RPE were found in both the present study and our previous work [22] whereas corticospinal excitability was increased, indicating that an increased internal sense of effort is not directly related to impaired corticospinal responsiveness. The awareness of the perception of effort may be built within the brain areas that are responsible for movement planning and control (i.e., frontal and prefrontal areas).

Similar to previous findings [22], [39], the different muscles of the *quadriceps femoris* did not behave identically in the present experiment. Indeed, RF and VL muscles tended to present similar MEP response patterns with fatigue but these were different than for VM. The different results between VL and VM are difficult to interpret because both muscles are monoarticular and their activation during cycling is believed to be similar. It is however possible that the intensity and/or the site of TMS was not optimal for the VM muscle since RF and VL muscles were the muscles of interest for the determination of the site and intensity of stimulation. This suggests that perhaps one muscle may not be used as a surrogate in determining *quadriceps femoris* changes in corticospinal excitability with fatigue.

#### Cortical silent periods

Similar to the aforementioned MEP responses, CSP duration generally increases with the development of fatigue following sustained isometric maximal and submaximal contractions (for a review, see reference [37]), including during lower-limb exercise. This is not the case in whole-body exercise [10], [21], [22], [36], [38], [39] and the present study confirms these findings. Again, differences between single-joint *vs.* whole-body exercise in terms of core temperature, blood glucose and muscle/cerebral oxygenation may explain these specific responses.

It has been argued that central effects of mechano- and metabo-sensitive group III and IV muscle afferents might facilitate the fatigue-induced increase in CSP during lower-limb [43] but not during upper-limb [15] exercise. Gruet et al. [37] suggested that similar to the differential influence of group III and IV afferents on the lower motoneurons innervating extensor and flexor muscles, it is possible that the role of these afferent fibers on fatigue-induced increases in intracortical inhibition depends on the investigated muscle group. Here, we are suggesting that it may also depend on the type of exercise, i.e. single-joint/isometric *vs.* whole-body/dynamic. One other explanation to partly explain the lack of CSP increase is the delay (∼3 min) between the end of exercise and the TMS measurements that allows the recovery of CSP duration to baseline values.

### Limits of the Study

The present experiment did not measure fatigue during exercise *per se*, nor right at exercise cessation since a short delay (∼3 min) was needed to install the subjects on the ergometer before testing. By measuring neuromuscular function regularly during an intense exercise performed on an isokinetic ergometer, we have recently shown that MVC and muscle contractile properties can recover quickly [32]. This is probably the main flaw of almost all whole-body exercise (cycling, running) studies, including the present one. The present exercise was a prolonged exercise, i.e. less intense than in Froyd et al. [32] so we may argue that recovery was likely less pronounced in a short period of time. However, future studies must measure neuromuscular function changes during whole-body exercise and immediately at exhaustion to better understand the factors causing fatigue and exhaustion.

In conclusion, the decrease in voluntary activation assessed by TMS validates the hypothesis that supraspinal fatigue occurs during prolonged cycling exercise. The present findings do not allow ascertaining whether this supraspinal fatigue is related to the implication of muscle afferent fibers or cerebral oxygen availability but do show that it is not related to reduced corticospinal excitability or elevated intra-cortical inhibition. This study also suggests that the complex interactions between peripheral failure and changes at the cortical/motoneuronal levels depend not only on the type of exercise (single-joint *vs.* whole-body) but also on exercise intensity and/or duration.
